# Aging clock based on nucleosome reorganisation derived from cell‐free DNA

**DOI:** 10.1111/acel.14100

**Published:** 2024-02-09

**Authors:** Mariya Shtumpf, Seihee Jeong, Milena Bikova, Hulkar Mamayusupova, Luminita Ruje, Vladimir B. Teif

**Affiliations:** ^1^ School of Life Sciences University of Essex Colchester UK

**Keywords:** aging, cell‐free DNA, cfDNA, liquid biopsy, NRL, nucleosome positioning, nucleosome repeat length, nucleosomics

## Abstract

Aging induces systematic changes in the distribution of nucleosomes, which affect gene expression programs. Here we reconstructed nucleosome maps based on cell‐free DNA (cfDNA) extracted from blood plasma using four cohorts of people of different ages. We show that nucleosomes tend to be separated by larger genomic distances in older people, and age correlates with the nucleosome repeat length (NRL). Furthermore, we developed the first aging clock based on cfDNA nucleosomics. Machine learning based on cfDNA distance distributions allowed predicting person's age with the median absolute error of 3–3.5 years.

## Graphical abstract

We show that aging is associated with an increase in the distance between nucleosomes, which can be used to predict a person's age and conduct age classification. We developed the first aging clock based on the nucleosomics analysis of cell‐free DNA (cfDNA) extracted from blood plasma. Machine learning based on cfDNA distance distributions enabled the prediction of a person's age with the median absolute error of 3–3.5 years.

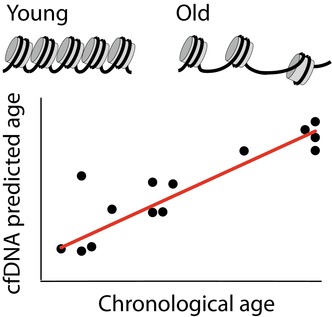

**Abbreviations**
bp: base pair
cfDNA: cell‐free DNA
NRL: nucleosome repeat length
PCA: principal component analysis
y.o.: years old

Genomic nucleosome positions are specific for a given cell and change over time. Since the 1970s, there have been a number of attempts to determine the change of distances between nucleosomes in human aging (Ishimi et al., 1987; Smith et al., 1989). This question is still open (Lu et al., 2021) and receiving increasing attention in current studies aiming to understand and increase longevity (Debes et al., 2023). Beyond its fundamental value, this question becomes important for new generations of medical diagnostics based on the analysis of cell‐free DNA (cfDNA). cfDNA mostly consists of nucleosome‐protected genomic regions undergoing digestion by nucleases (Shtumpf et al., 2022; Snyder et al., 2016). It has been shown recently that the nucleosome repeat length (NRL) decreases in some cancers and can be used for patient stratification (Jacob et al., 2023; Piroeva et al., 2023). Therefore, it is critical to know whether age‐associated nucleosome rearrangement has systematic trends, and whether these go in the same or opposite direction to the trends associated with cancer. To resolve this challenge, we used here cfDNA from four cohorts of people of different ages (Cristiano et al., 2019; Peneder et al., 2021; Razavi et al., 2019; Teo et al., 2019).

First, we analysed a relatively small data set of Teo et al. (2019) comprising of 12 people of three age groups: 25, 70 and 100 years old (y.o.). We compared pairwise cfDNA occupancy landscapes in 25‐ and 100‐year olds and determined 100‐bp regions with significantly increased cfDNA occupancy in centenarians versus 25 year olds. The normalised cfDNA occupancy scores in these regions were used to cluster samples using principal component analysis (PCA) (Figure 1a). People who are 70 y.o. were not included in the PCA discovery data set, but these were clearly positioned along the principal component PC1, separately from 25 to 100 y.o. We noted that a single principal component PC1 accounted for about half of the variation (49% in the case of the Teo et al. cohort). A similar situation was observed using a larger cohort from Cristiano et al. (Cristiano et al., 2019) which contains 79 deep‐sequenced cfDNA samples, where 53% variance in age between groups of ≤40 and ≥70 y.o. healthy people was explained by a single principal component PC1 (Figure 1b).

**FIGURE 1:**
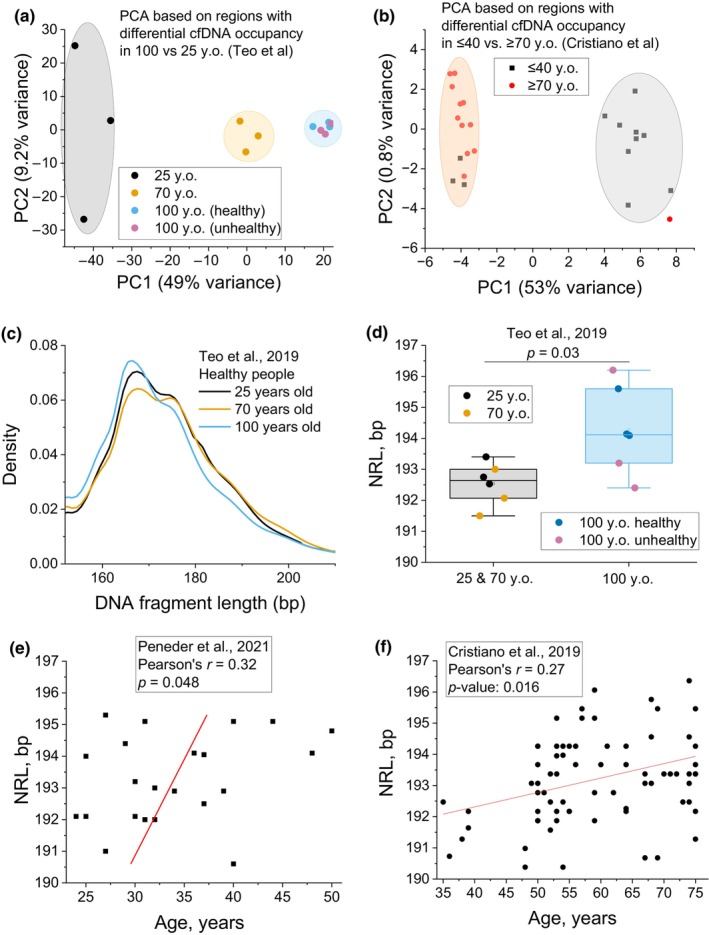
Differences in cfDNA reflect healthy aging. (a) Principal component analysis (PCA) allows stratification of ages in the cohort of Teo et al. (2019) based on cfDNA occupancy in regions which gained nucleosomes in 100 y.o. people in comparison with 25 y.o. Healthy people with ages 25 (black), 70 (orange) and 100 y.o. (blue), as well as unhealthy 100 y.o. (violet) (Teo et al.). (b) PCA based on genome‐wide regions with differential nucleosome occupancy between age groups ≤40 and ≥70 y.o. for healthy female from the cohort of Cristiano et al. (2019). (c) Distribution of cfDNA fragment sizes among different age groups in the cohort of Teo et al. (d) NRLs for the cohort of Teo et al. for each person (circles), group‐average values (open squares), medians (horizontal lines) and variance intervals (filled bars). (e and f) Correlation of NRL and age for the cohort of Peneder et al. (2021) (e) and Cristiano et al. (f).

What is the biological meaning of a single variable associated with nucleosome maps explaining a large variation in age? There are only a few classical characteristics of nucleosome positioning that can be represented by a single number. One of such integral characteristics is the distribution of cfDNA fragment sizes, which is usually dominated by chromatosome‐size fragments in healthy people and become shorter in cancer (Underhill et al., 2016). In the cohort of Teo et al., the distribution of fragment sizes was slightly shifted to shorter sizes for centenarians, but this was not significant for this small cohort of 12 people (Figure 1c). Another integral nucleosome characteristic is the NRL. Our analysis shows that the NRL in centenarians was ~1.5 bp larger than that in young and middle‐aged people (paired‐sample *t*‐test *p* = 0.03), and more heterogeneous (Figure 1d). For comparison, NRL difference between stem cells and differentiated fibroblasts is ~5 bp, which corresponds to dramatic changes in 3D chromatin properties (Teif et al., 2012). We also confirmed the correlation between age and NRL using larger cohorts of Peneder et al. (2021) (22 deep‐sequenced cfDNA samples, Pearson's *r* = 0.32; *p* = 0.048, Figure 1e) and Cristiano et al. (2019) (79 deep‐sequenced cfDNA samples, Pearson's *r* = 0.27; *p* = 0.016, Figure 1f). Combining the latter cohort with additional data set from Razavi et al. (2019) (24 deep‐sequenced samples) further decreased *p*‐value down to <0.0001 (Figure S13). Thus, studies in each of these four cohorts confirm that older people tend to have longer NRL. It is important to note that our previous finding of NRL *decrease* in cancer patients (Jacob et al., 2023; Piroeva et al., 2023) represents an opposite trend, which offers a possibility to uncouple the effects of aging and cancer while performing nucleosomics‐based diagnostics based on cfDNA. In terms of possible mechanisms, if shorter NRL in cancer is a feature of actively dividing cells, longer NRL in aging may reflect decreased gene activity with age.

Next, we asked whether nucleosome positioning reconstructed from cfDNA can be used to predict a person's age in analogy to the DNA methylation age clock (Horvath & Raj, 2018; Simpson & Chandra, 2021). To check this hypothesis, we used deep‐sequenced cfDNA samples from Cristiano et al. (2019), and constructed age predictors separately based on the DNA fragment size distribution and the autocorrelation function of the distances between nucleosomes (nucleosome–nucleosome distances defined by centres of cfDNA fragments). Figures 2a,b visualise these two types of distributions averaged across the groups of <40 y.o and >70 y.o. These average profiles indicate a difference in the fine‐scale chromatin organisation between these age groups—older people have a smaller proportion of dinucleosomes and trinucleosomes in comparison with younger people. We then constructed a multiple linear regression machine learning model to predict a person's age based on each of these two types of distributions (See Supplementary Methods). Both these predictors had similarly good performance (Pearson's correlation between predicted and chronological ages *r* ≈ 0.85, MSE ≈ 34), as detailed in Figure 2c,d and Supplementary Methods. The median absolute error for cfDNA‐based aging clock was 3.0 and 3.5 years for our models in Figure 2c,d correspondingly, which is comparable to methylation‐based clocks, and significantly outperforms aging clocks based on chromatin accessibility in blood cells (Morandini et al., 2023). Furthermore, the advantage of cfDNA‐based aging clock described here is that it does not require experiments in specific cells. We have also checked that our method allows the classification of samples based on the age group, for example, the split into group ≤55 y.o. and >55 y.o. can be achieved with AUC = 0.96 and 0.93 using predictors based on nucleosome–nucleosome distances and cfDNA fragment sizes correspondingly (see Supplementary Methods and Figure S14).

**FIGURE 2:**
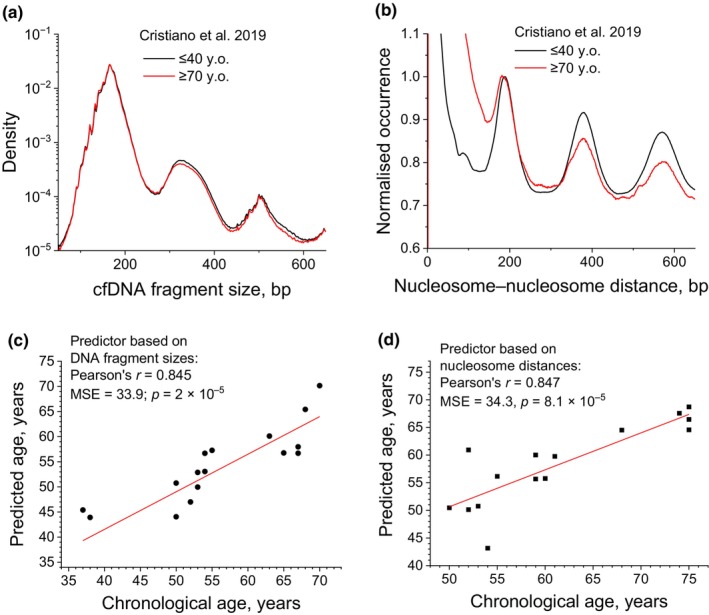
Construction of aging clocks based on nucleosomics of cfDNA. (a) Averaged distributions of cfDNA fragment sizes for people ≤40 y.o. (black) and ≥70 y.o. (red). (b) Autocorrelation of distances between nucleosomes represented by centres of cfDNA fragments (≤40 y.o., black; ≥70 y.o., red). (c) Age prediction using linear regression model based on cfDNA fragment size distributions. (d) Age prediction using linear regression model based on distances between nucleosomes. The analyses are based on deep‐sequenced cfDNA samples from Cristiano et al. (2019) by splitting the data into 80% training and 20% testing.

To summarise, our results show that older people tend to have longer genomic distances between nucleosomes and that with age the amount of di‐ and tri‐nucleosomes protected from digestion decreases. Furthermore, we showed that nucleosomics analysis of cfDNA can stratify people by age and predict a person's age with reasonable precision. The nucleosomics‐based aging clock can be a valuable tool on its own and may be also relevant for nucleosomics analyses in patient diagnostics with liquid biopsies based on cfDNA (Jacob et al., 2023; Piroeva et al., 2023).

## METHODS

For the data set of Teo et al. (2019), sequenced cfDNA in nine healthy individuals with ages 25, 70 and 100 y.o. as well as three unhealthy centenarians was downloaded from GEO (GSE114511). For the data sets of Peneder et al. (2021), Cristiano et al. (2019) and Razavi et al. (2019), controlled access data were downloaded from EGA entries EGAS00001005127, EGAD00001005339 and EGAD00001005302 correspondingly. The Peneder et al. data set contained 22 deep‐sequenced cfDNA samples from healthy people. The Cristiano et al. data set contained samples with different sequencing coverage, of which we selected 79 cfDNA samples from healthy people with sequencing coverage >30 million mapped paired‐end reads that allowed robust determination of NRL (Figure 1f). In addition, we performed a similar analysis with the data set of Razavi et al. using 24 samples from healthy people where age was identifiable (Table S3). Paired‐end reads were aligned to human genome hg19 using Bowtie2 (Langmead & Salzberg, 2012) accepting uniquely mappable reads with up to two mismatches, then processed with NucTools (Vainshtein et al., 2017) to generate BED files with genomic coordinates and cfDNA fragment sizes. NRLs were determined with NucTools (Vainshtein et al., 2017) and NRLcalc (Clarkson et al., 2019) as described previously (Piroeva et al., 2023). Figures S1–S12 exemplify NRL calculations for the Teo et al. data set. NRL values calculated for the data sets of Cristiano et al., Peneder et al. and Razavi et al. are listed in Tables S1–S3. To stratify ages with PCA based on differential nucleosome occupancy, we performed a procedure as in Piroeva et al., (2023), detailed in Supplementary Methods, and the genomic regions used in this analysis are listed in Tables S4–S6. The aging clock supported by machine learning (ML) was developed using two types of linear regression ML models based on the concept of differential nucleosome spacing, as represented by Figure 2c,d. The ML approaches for age stratification and prediction are detailed in Supplementary Methods.

## AUTHOR CONTRIBUTIONS

Machine learning: SJ; Bioinformatics analysis: MS, MB, HM, LR and VBT; Study design, methodology, supervision and manuscript preparation: VBT.

## CONFLICT OF INTEREST STATEMENT

The authors declare no conflict of interest.

## Supporting information


Data S1

Tables S4–S6

## Data Availability

The data set of Teo et al. is available in GEO (GSE114511). The data sets of Peneder et al., Cristiano et al. and Razavi et al. are available in EGA (EGAS00001005127, EGAD00001005339 and EGAD00001005302 correspondingly).
