# Supplementary material for: Aging clock based on nucleosome reorganisation derived from cell‐free DNA
Source: Aging Cell. 2024 Feb 9;23(5):e14100. doi: 10.1111/acel.14100 (PMC11113261; doi:10.1111/acel.14100)
Supplement: Supplementary file 1 — Data S1 [file ACEL-23-e14100-s002.pdf]

Supplementary Materials

Aging clock based on nucleosome reorganisation derived from cell-free DNA

Mariya Shtumpf, Seihee Jeong, Milena Bikova, Hulkar Mamayusupova, Luminita Ruje and Vladimir B. Teif*

School of Life Sciences, University of Essex, Wivenhoe Park, Colchester, CO4 3SQ, UK

* Correspondence should be addressed to Vladimir B Teif (vteif@essex.ac.uk).

1. Supplementary Methods

PCA analysis. PCA analysis was performed following the workflow described previously (Piroeva et al., 2023). We calculated the normalised cfDNA occupancy for each sample for each 100-bp genomic region and calculating the average value per window per sample. Next, we determined "stable-nucleosome" 100-bp regions where the relative variation of cfDNA occupancy was < 0.5 across all samples of a given condition (age group). Then we performed a pairwise comparison of the condition-average cfDNA profiles. In the case of the Teo et al cohort, we used 25 y.o. and 100 y.o. as two age groups to determine 100-bp regions with differential nucleosome occupancy on chromosome 1 where the relative change of normalised cfDNA occupancy in centenarians versus 25 years olds was > 0.95. The normalised cfDNA occupancy values of individual samples inside these regions were used to perform PCA, including in this analysis samples from 70 y.o. which were not used in the original construction of these regions. The coordinates of genomic regions used for the PCA analyses in Figure 1A are provided in Supplementary Tables S4. About 5% of these regions were located within +/-1000 base pairs from annotated transcription start sites (Supplementary Table S5). In the case of the Cristiano et al cohort, we applied a similar procedure, using deep-sequenced samples for healthy female donors with ages ≤ 40 y.o. and ≥70 y.o., selecting genomic regions for PCA analysis across all chromosomes, where relative nucleosome occupancy increased above a threshold of 0.4, and where the region was quantified with non-zero nucleosome occupancy in all 174 healthy female samples from

the Cristiano cohort, including samples with low sequencing coverage to create a more robust classifier (Supplementary Table S6). The latter regions were located outside of annotated GRCh37 genes. The manipulations with BED files were performed using BedTools (Quinlan & Hall, 2010). PCA was calculated using R.

Age prediction based on nucleosome-nucleosome distances. Two types of machine learning (ML) models were constructed, as represented by Figures 2C and 2D. The age prediction model was developed by training a linear regression algorithm on 80% of the dataset. This approach assumes a linear relationship between the biological age of the donors and selected features based either on the distribution of nucleosome-nucleosome distances (Figure 2D) or cfDNA fragment sizes (Figure 2C). For the model based on nucleosome-nucleosome distances (Figure 2D), the distributions as in Figure 2B were calculated for each individual sample in the range [50bp, 2000bp] and each sample was normalised by subtracting the mean and dividing by the maximum value of the Y-coordinate within that sample. To address the high dimensionality inherent in our dataset, we employed a feature selection strategy based on the identification of the peaks of the nucleosome-nucleosome distance distributions. The median over density values at each nucleosome-nucleosome distance was calculated across all Christiano et al. samples with high sequencing coverage used in the analysis, and the Savitzky-Golay filter with a polynomial order of 2 and a window size of 123 was applied to smooth the distribution. Subsequent to smoothing, we identified local maxima, using the *find_peaks* method from the *Scipy* Python library, employing a threshold of 0.000001 for the relative difference in the normalised Y-coordinate values and a minimal distance between two features of 90 bp. This yielded a set of 10 peaks, which were further refined with *SelectKBest* feature selection method from sklearn Python library based on the ANOVA F-statistic, upon which one of the features was excluded. Then the linear regression function of the *sklearn* library was applied using these 9 features.

Age prediction based on cfDNA fragment size distributions. For ML model based on cfDNA fragment sizes (Figure 2C), the procedure described above was applied with the following modifications. The input data was in the form of the density of fragment sizes as in Figure 2B, calculated separately for each sample using data from 85 healthy people reported by Cristiano et al. Using the mean over density values at each fragment size, we applied a Gaussian filter requiring a minimal distance between two peaks of 45 bp and a threshold of 0.0000001 for the relative difference in Y-coordinate values and determined the local maxima on this curve using the *find_peaks* method from the Scipy Python library. This resulted in 33 features, which were further refined with *SelectKBest* feature selection method from *sklearn* Python library based on ANOVA F-statistic, upon which one of the features was

excluded. Then the linear regression function of *sklearn* was applied using these features. For both ML models the data was randomly split into training and testing as 80% and 20% correspondingly. The performance of ML models was evaluated using mean squared error (MSE) and Pearson correlation (r).

Age classification using ML. We have created ML models that classify age into the following two groups: 1) ≤ 55 y.o. 2) > 55. This selection of ages was made in order to have a balanced number of samples for each age group based on the available age distribution in the Cristiano et al dataset. Similar to age prediction using linear regression, we have constructed two models, based on cfDNA fragment sizes, and based on nucleosome-nucleosome distances. The data preprocessing was done in the same way as for the linear regression ML models described above. For the classification model based on nucleosome-nucleosome distances, we identified 9 significant peaks of the distribution. The normalized y-coordinates of these peaks were used as features in a logistic regression algorithm. We employed the logistic regression algorithm from the scikit-learn library, with the following parameters: regularization strength (C) set to 1000, the solver as 'newton-cg', the penalty as 'l2', and the maximum number of iterations (max_iter) set to 1000. This model achieved F1 = 0.9 and AUC = 0.96. For the predictor based on cfDNA fragment sizes, the classification model retained the same data preprocessing, but a different smoothing parameter of sigma 2.2 was applied which yielded 6 peaks of the fragment size distribution. Moreover, we have deployed the Random Forest algorithm implemented in the scikit-learn library, with hyperparameters configured as follows: min_samples_leaf=1, min_samples_split=2, max_depth set to unlimited, and a total of 100 trees (n_estimators=100). The model achieved F1 = 0.91 and AUC score of 0.93.

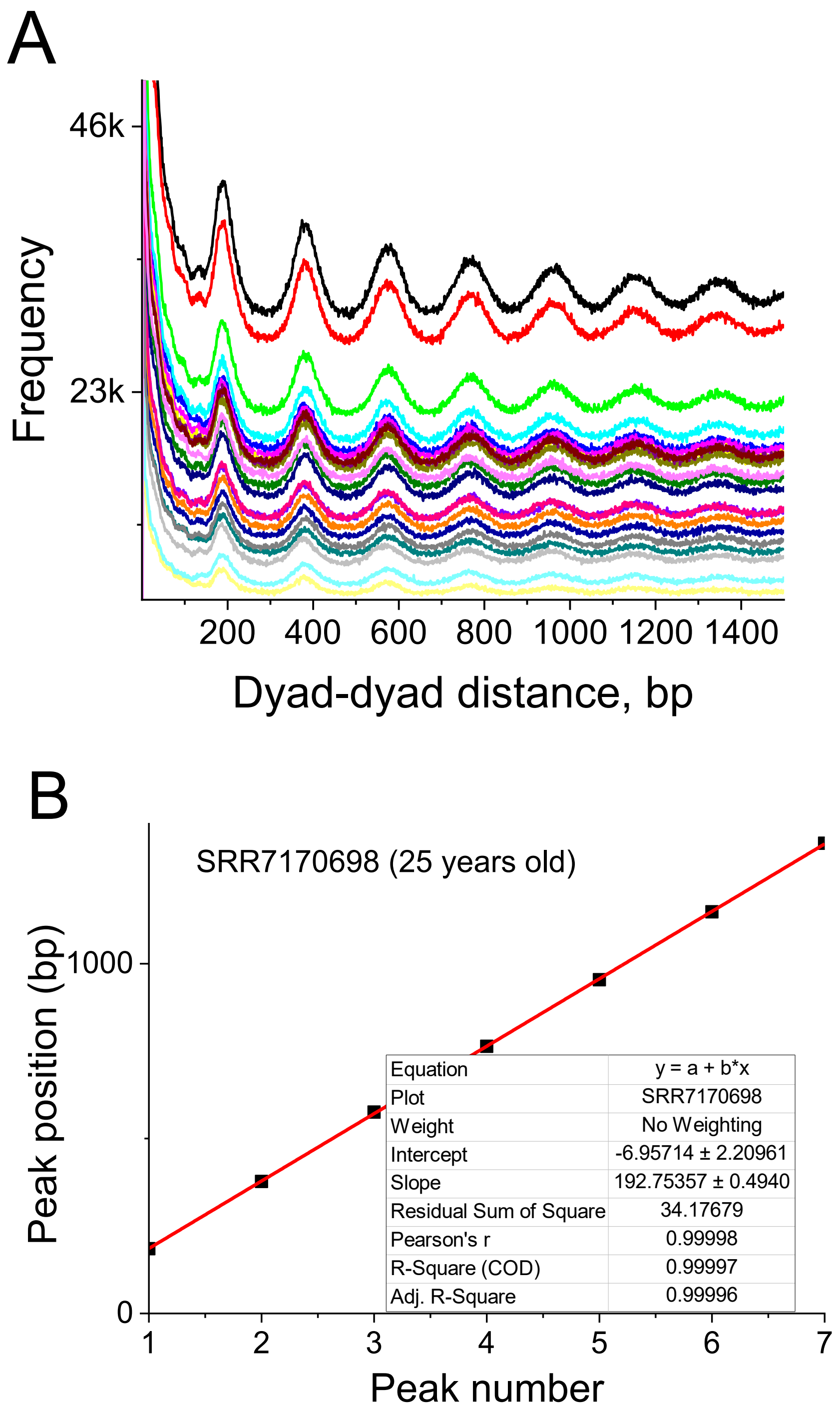

Supplementary Figure S1. A) The distribution of distances between centers of cfDNA fragments calculated with NucTools for cfDNA sample from a 25 years old person (SRA accession number SRR7170698), shown separately for each chromosome. B) Linear regression of the locations of the peak summits of the average genome-wide profile of the distribution of distances between centers of cfDNA fragments from (A). The slope of the linear fit line is equal to the nucleosome repeat length value.

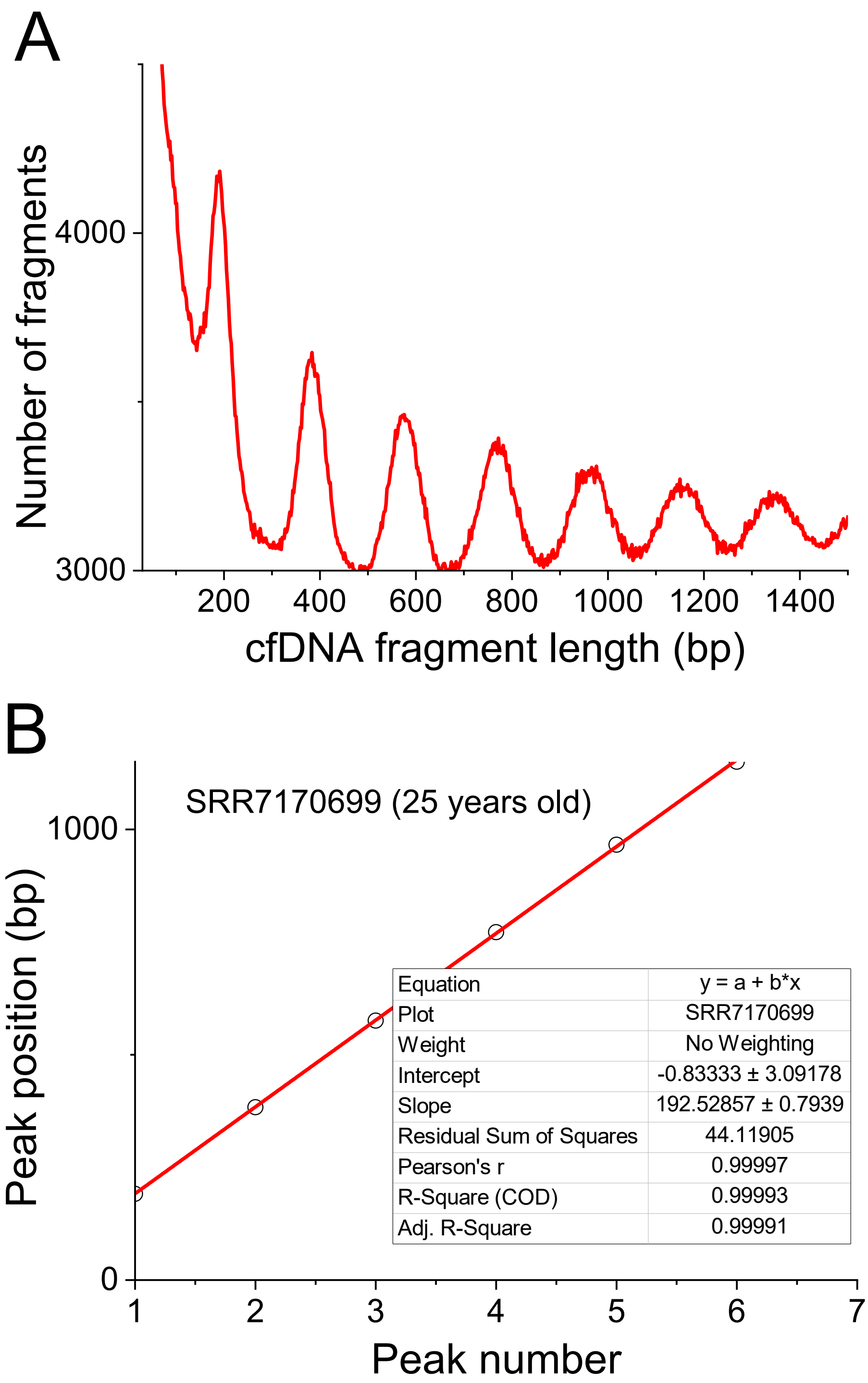

Supplementary Figure S2. A) The distribution of distances between centers of cfDNA fragments calculated with NucTools for cfDNA sample from a 25 years old person (SRA accession number SRR7170699), averaged across all chromosomes. B) Linear regression of the locations of the peak summits of the average genome-wide profile of the distribution of distances between centers of cfDNA fragments from (A). The slope of the linear fit line is equal to the nucleosome repeat length value.

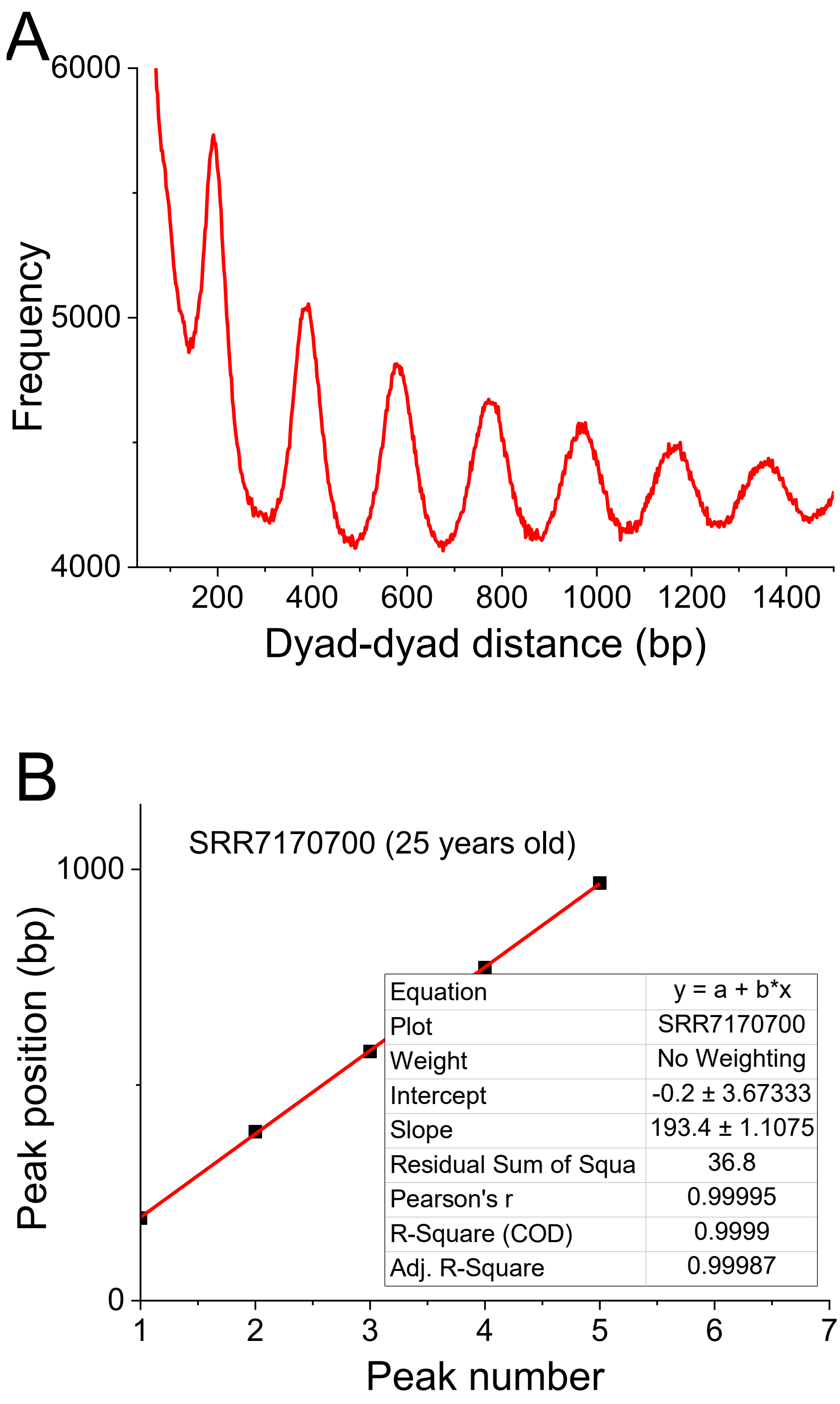

Supplementary Figure S3. A) The distribution of distances between centers of cfDNA fragments calculated with NucTools for cfDNA sample from a 25 years old person (SRA accession number SRR7170700), averaged across all chromosomes. B) Linear regression of the locations of the peak summits of the average genome-wide profile of the distribution of distances between centers of cfDNA fragments from (A). The slope of the linear fit line is equal to the nucleosome repeat length value.

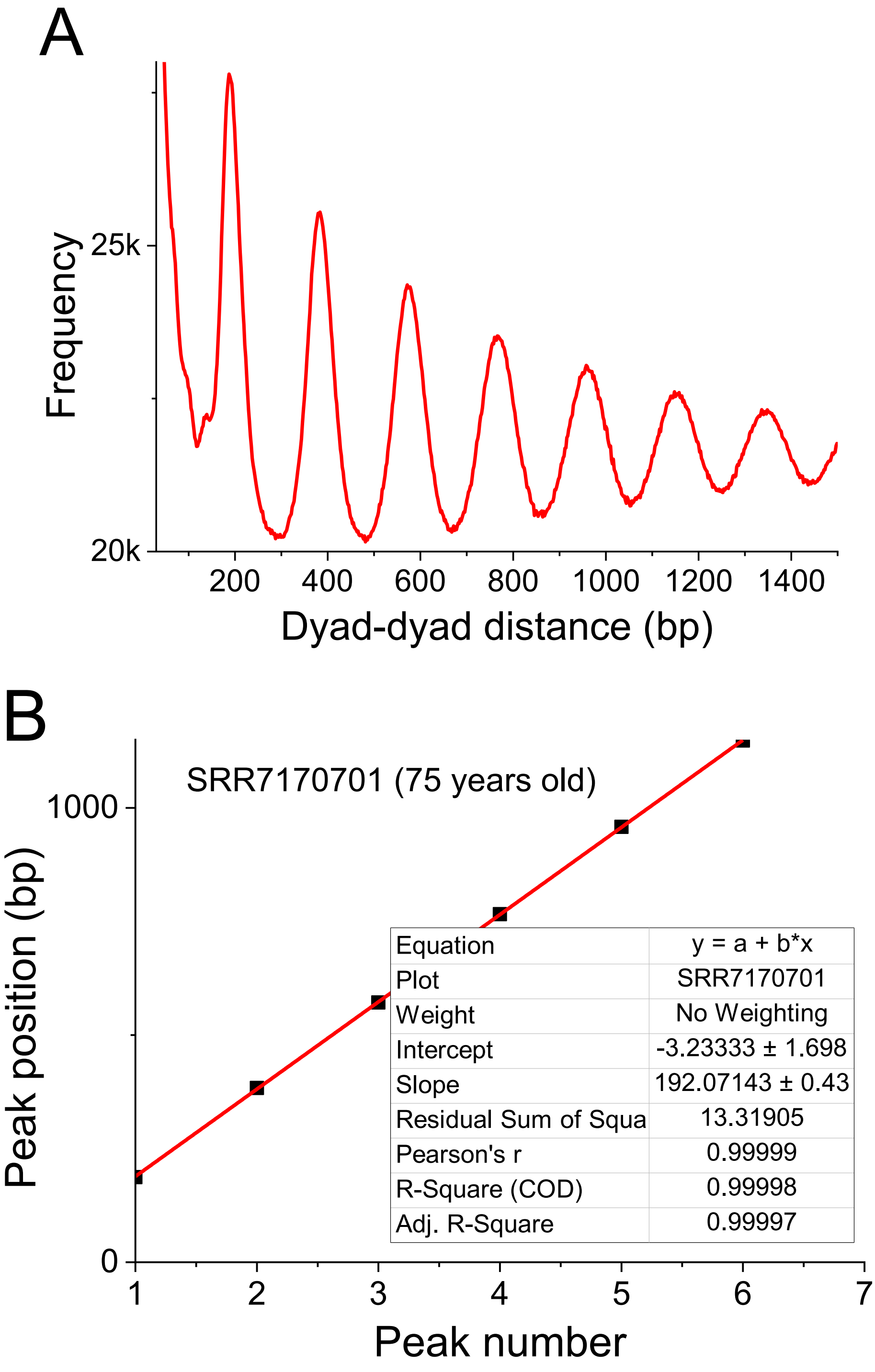

Supplementary Figure S4. A) The distribution of distances between centers of cfDNA fragments calculated with NucTools for cfDNA sample from a 75 years old person (SRA accession number SRR7170701), averaged across all chromosomes. B) Linear regression of the locations of the peak summits of the average genome-wide profile of the distribution of distances between centers of cfDNA fragments from (A). The slope of the linear fit line is equal to the nucleosome repeat length value.

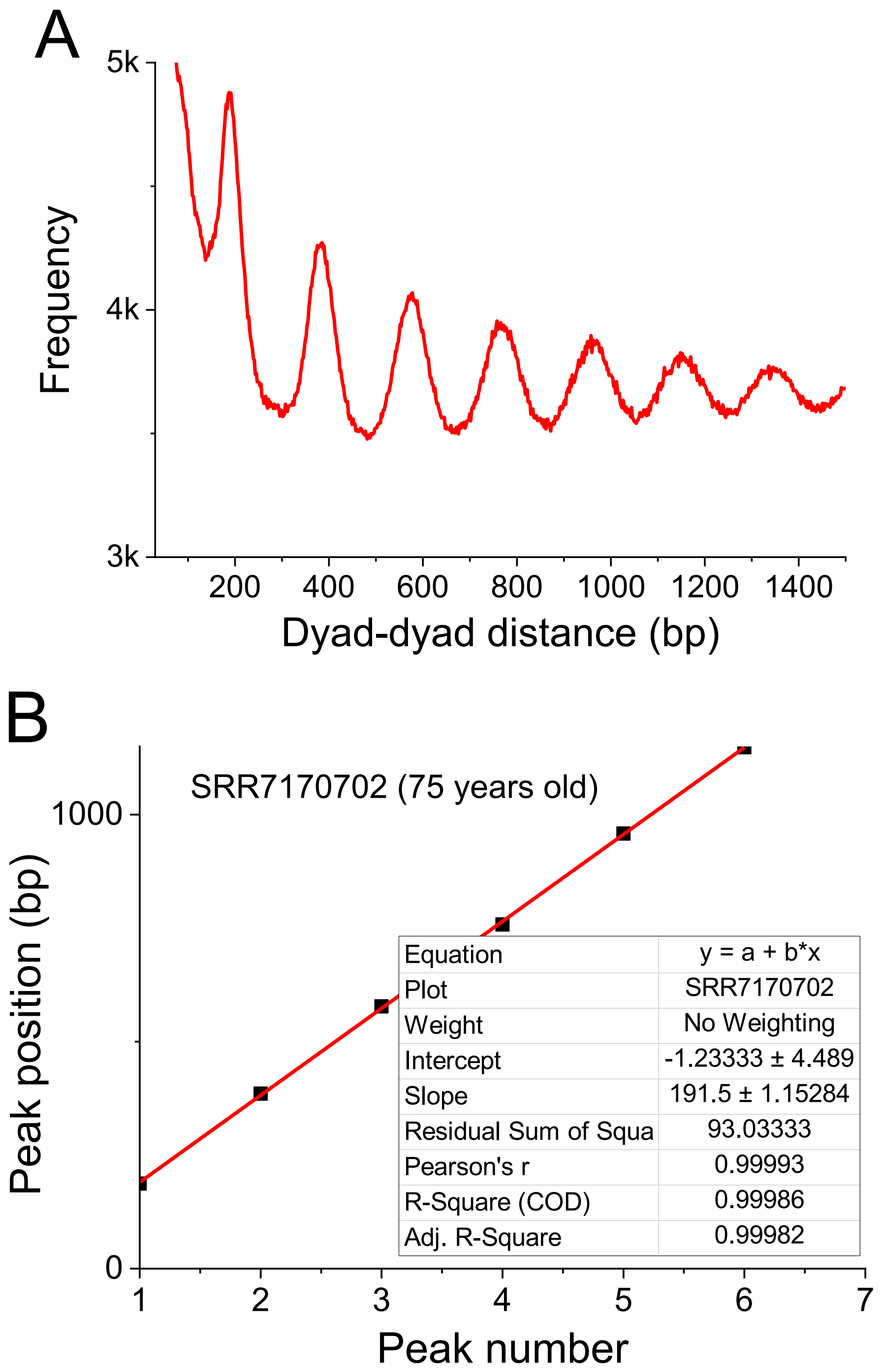

Supplementary Figure S5. A) The distribution of distances between centers of cfDNA fragments calculated with NucTools for cfDNA sample from a 75 years old person (SRA accession number SRR7170702), averaged across all chromosomes. B) Linear regression of the locations of the peak summits of the average genome-wide profile of the distribution of distances between centers of cfDNA fragments from (A). The slope of the linear fit line is equal to the nucleosome repeat length value.

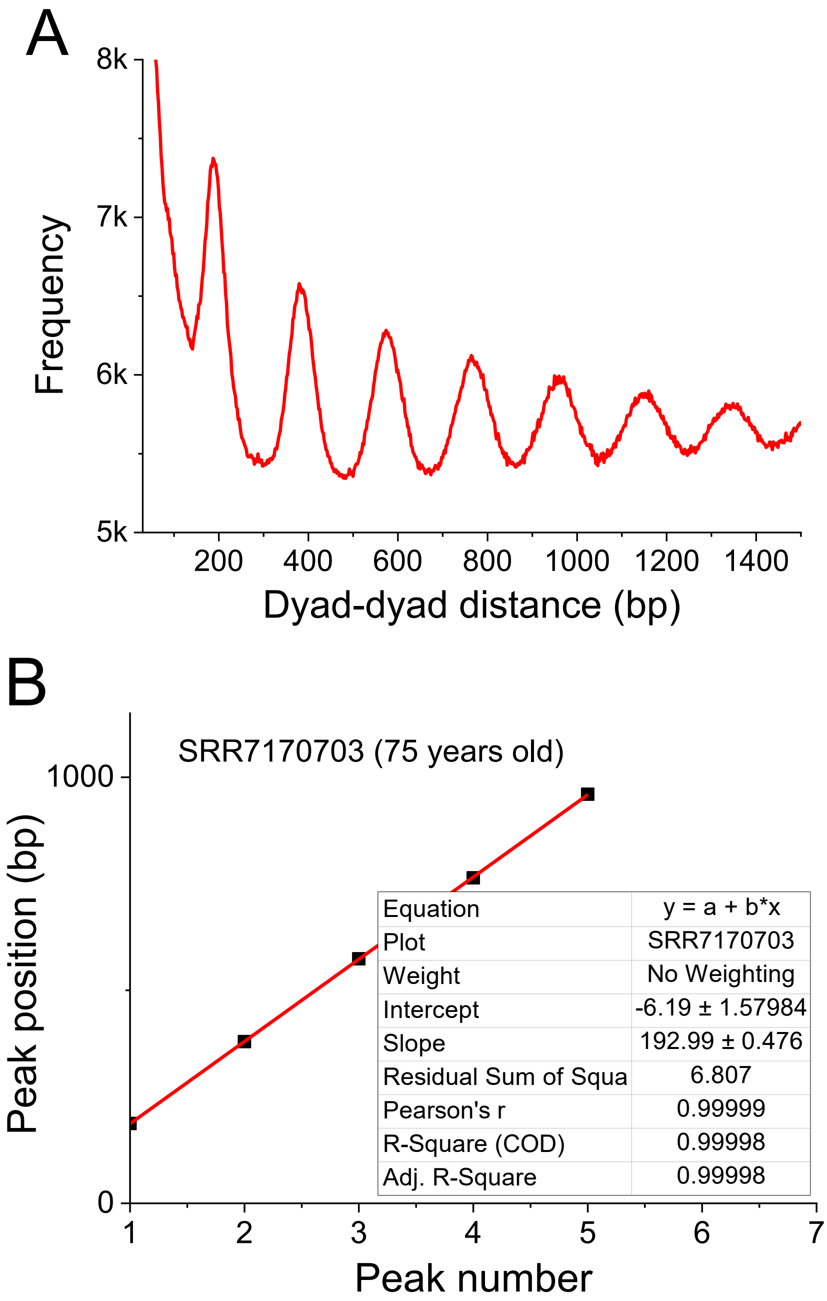

Supplementary Figure S6. A) The distribution of distances between centers of cfDNA fragments calculated with NucTools for cfDNA sample from a 75 years old person (SRA accession number SRR7170703), averaged across all chromosomes. B) Linear regression of the locations of the peak summits of the average genome-wide profile of the distribution of distances between centers of cfDNA fragments from (A). The slope of the linear fit line is equal to the nucleosome repeat length value.

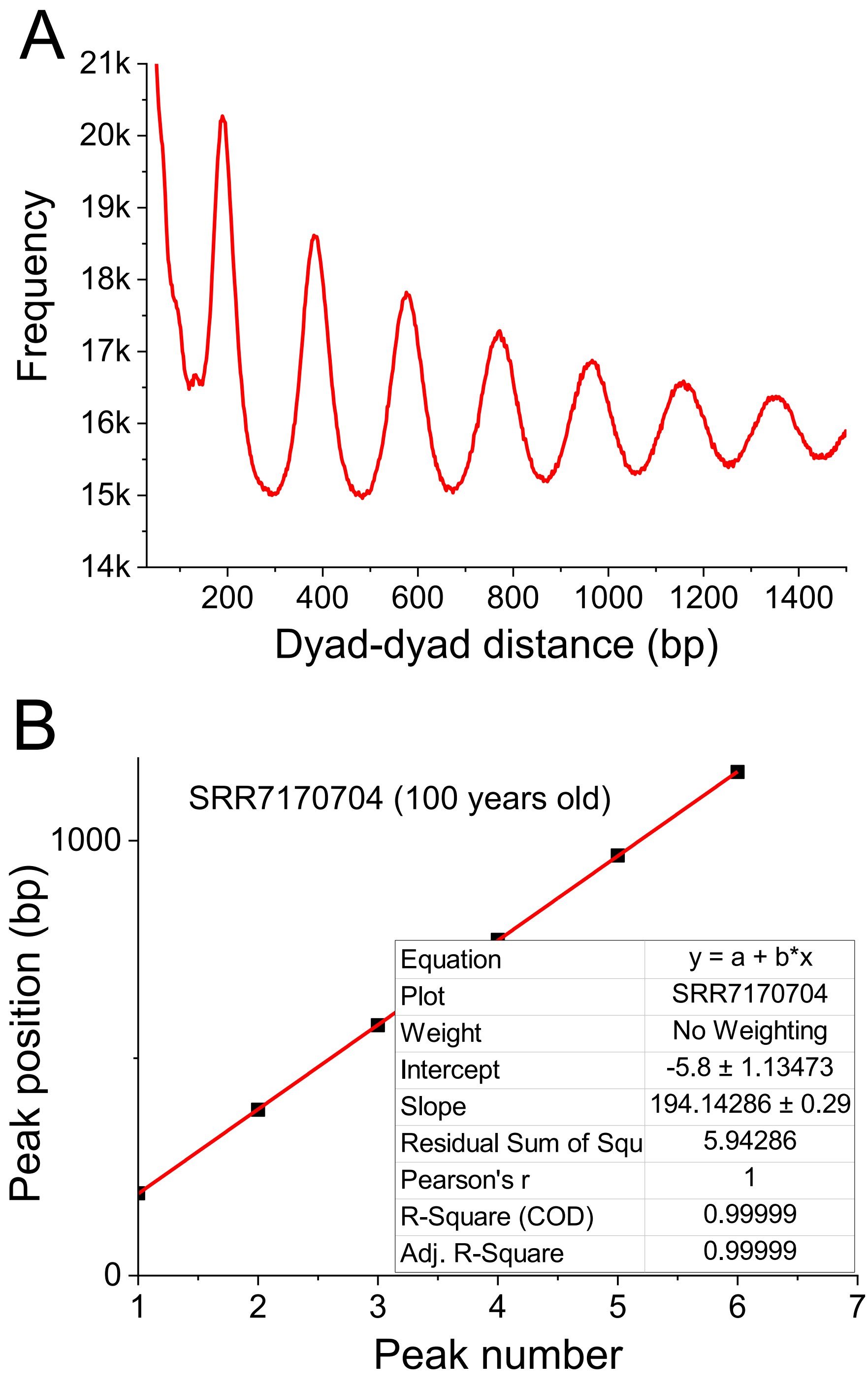

Supplementary Figure S7. A) The distribution of distances between centers of cfDNA fragments calculated with NucTools for cfDNA sample from a 100 years old person (SRA accession number SRR7170704), averaged across all chromosomes. B) Linear regression of the locations of the peak summits of the average genome-wide profile of the distribution of distances between centers of cfDNA fragments from (A). The slope of the linear fit line is equal to the nucleosome repeat length value.

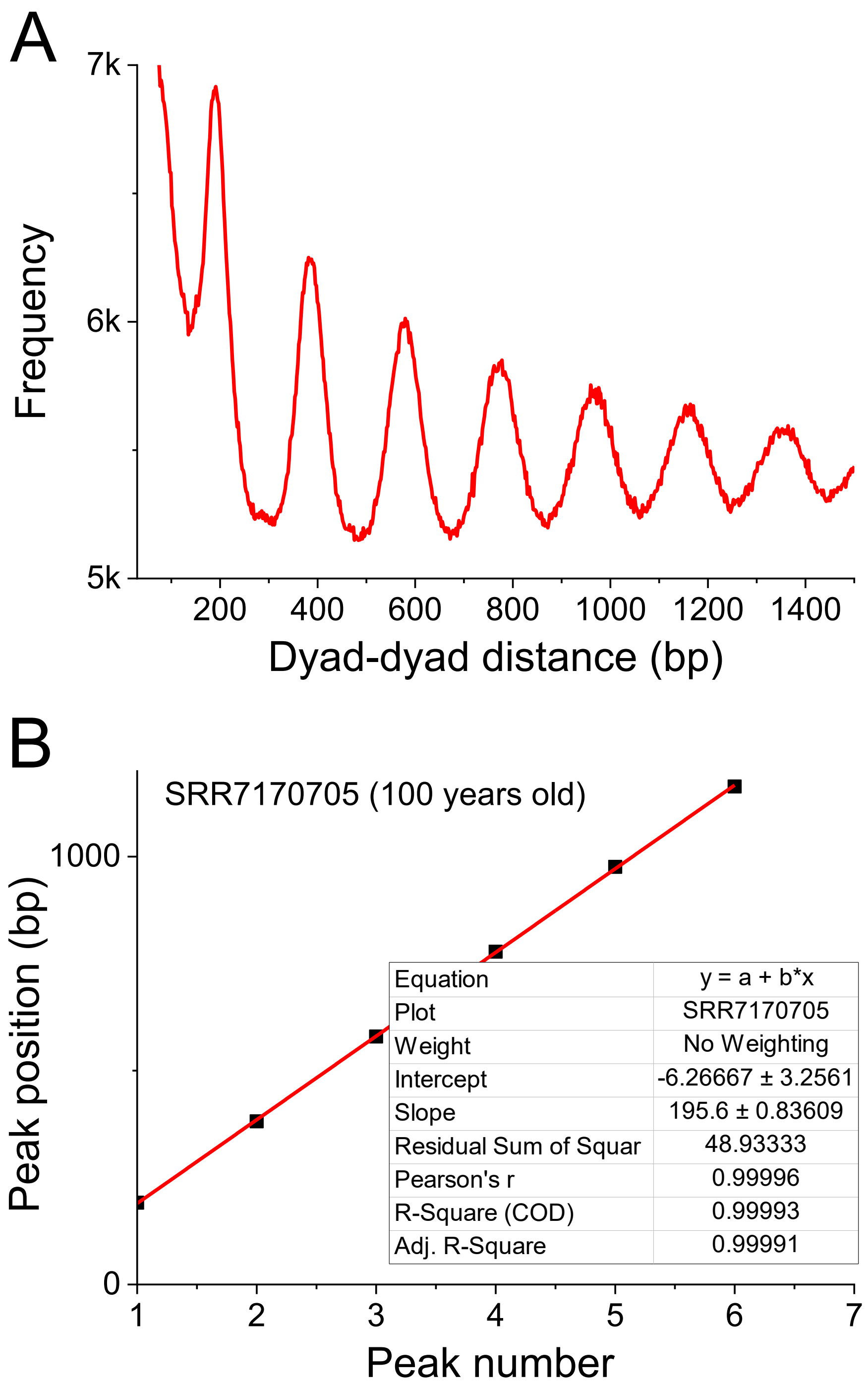

Supplementary Figure S8. A) The distribution of distances between centers of cfDNA fragments calculated with NucTools for cfDNA sample from a 100 years old person (SRA accession number SRR7170705), averaged across all chromosomes. B) Linear regression of the locations of the peak summits of the average genome-wide profile of the distribution of distances between centers of cfDNA fragments from (A). The slope of the linear fit line is equal to the nucleosome repeat length value.

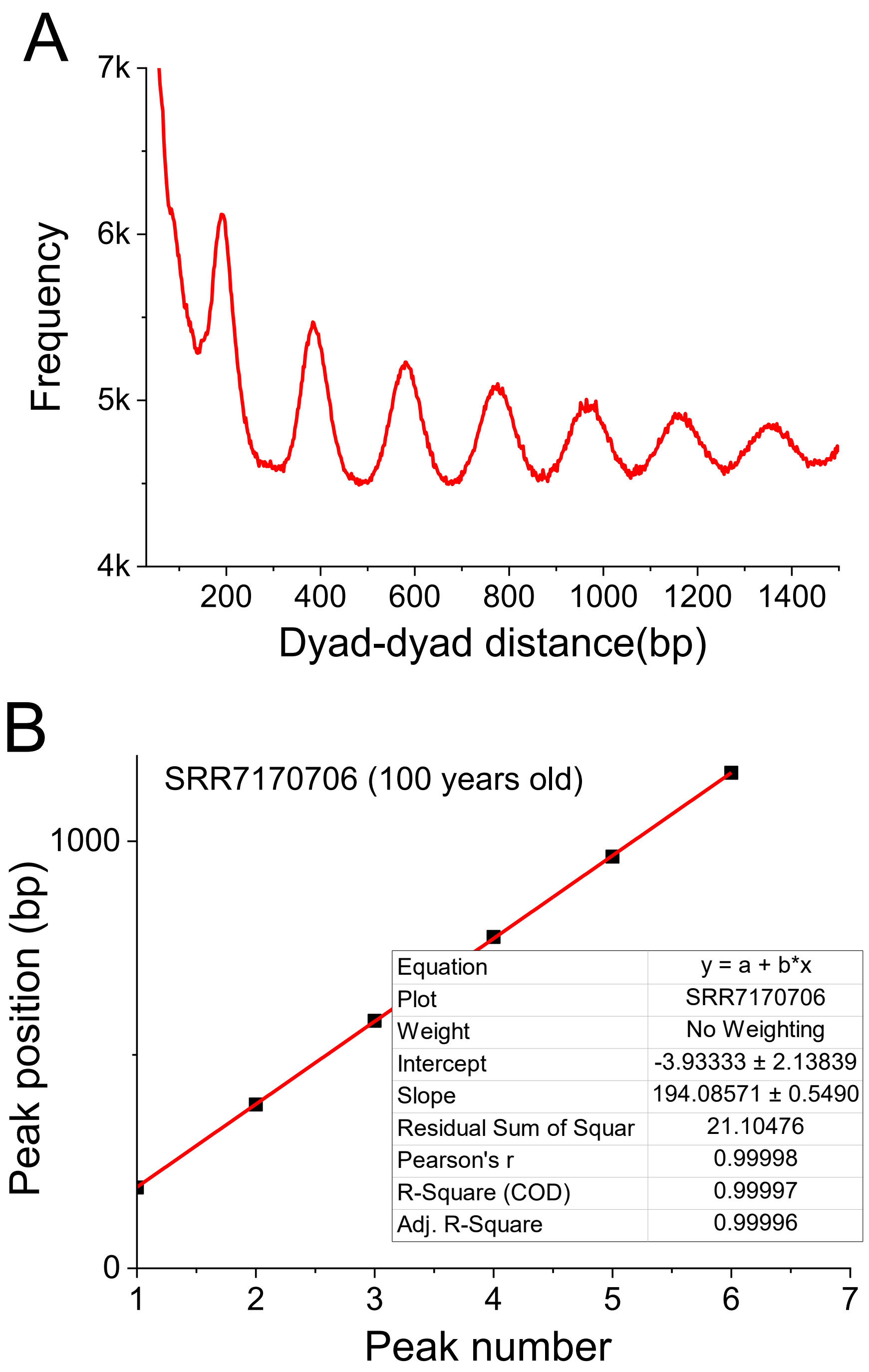

Supplementary Figure S9. A) The distribution of distances between centers of cfDNA fragments calculated with NucTools for cfDNA sample from a 100 years old person (SRA accession number SRR7170706), averaged across all chromosomes. B) Linear regression of the locations of the peak summits of the average genome-wide profile of the distribution of distances between centers of cfDNA fragments from (A). The slope of the linear fit line is equal to the nucleosome repeat length value.

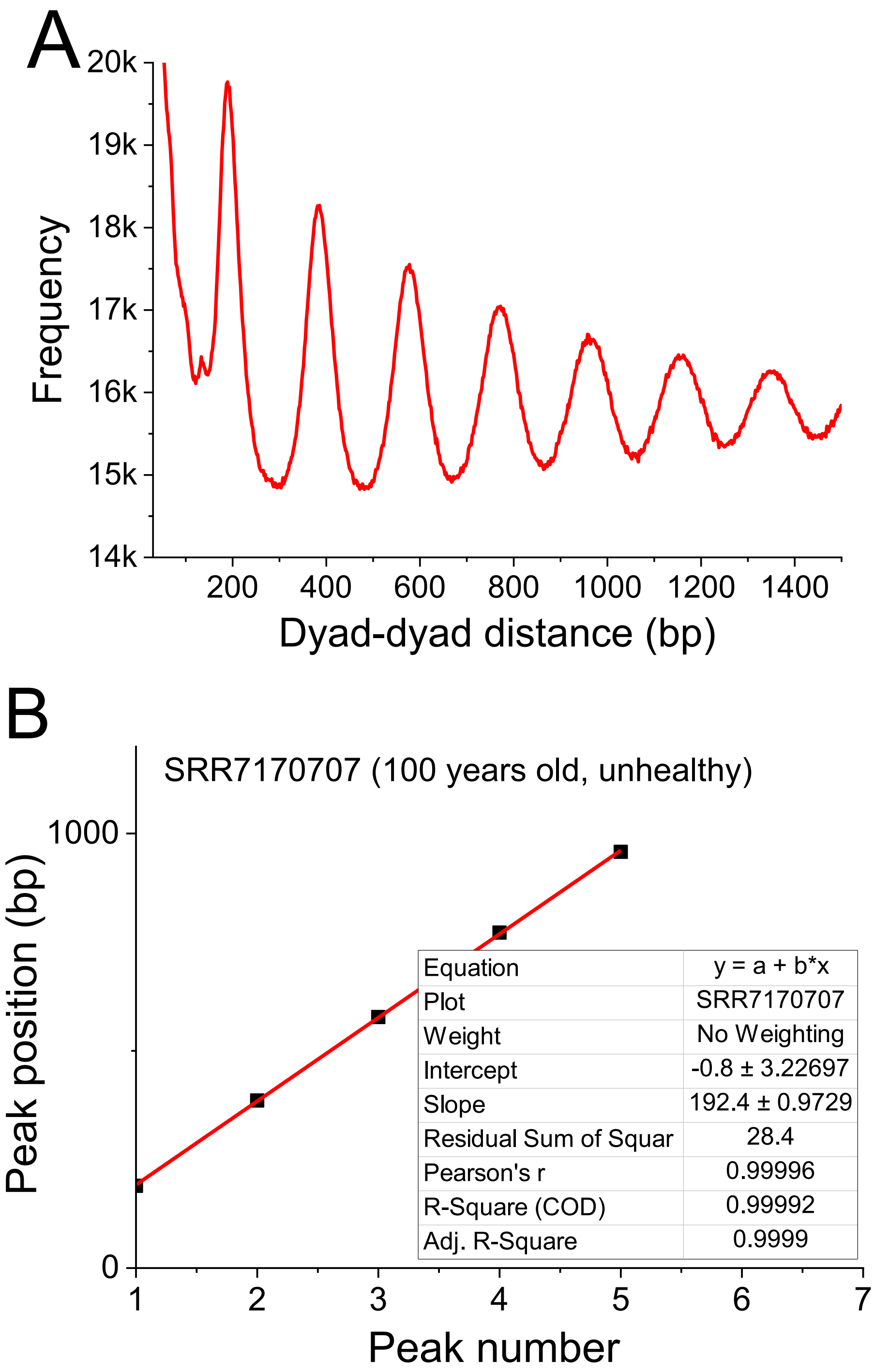

Supplementary Figure S10. A) The distribution of distances between centers of cfDNA fragments calculated with NucTools for cfDNA sample from a 100 years old person (SRA accession number SRR7170707), averaged across all chromosomes. B) Linear regression of the locations of the peak summits of the average genome-wide profile of the distribution of distances between centers of cfDNA fragments from (A). The slope of the linear fit line is equal to the nucleosome repeat length value.

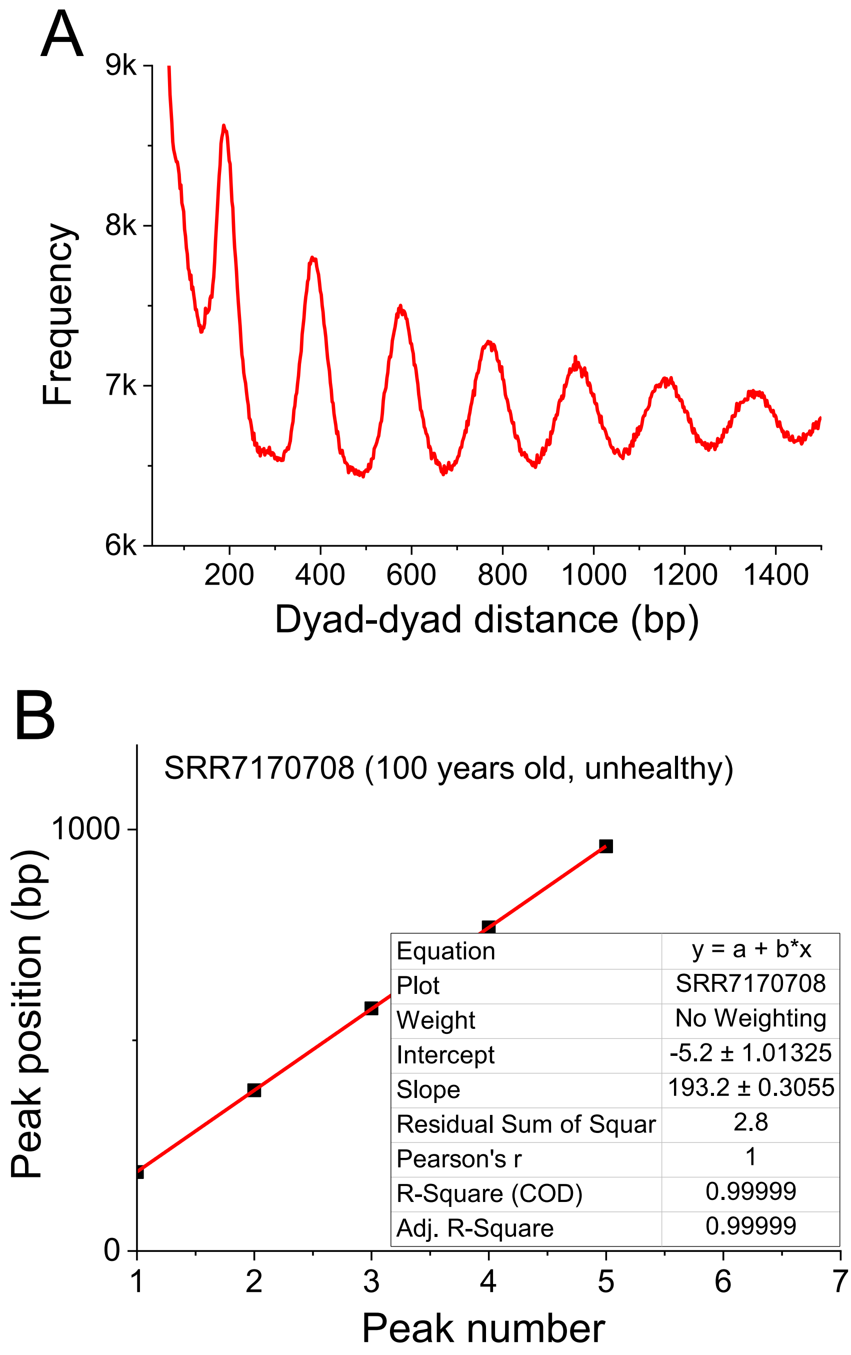

Supplementary Figure S11. A) The distribution of distances between centers of cfDNA fragments calculated with NucTools for cfDNA sample from a 100 years old person (SRA accession number SRR7170708), averaged across all chromosomes. B) Linear regression of the locations of the peak summits of the average genome-wide profile of the distribution of distances between centers of cfDNA fragments from (A). The slope of the linear fit line is equal to the nucleosome repeat length value.

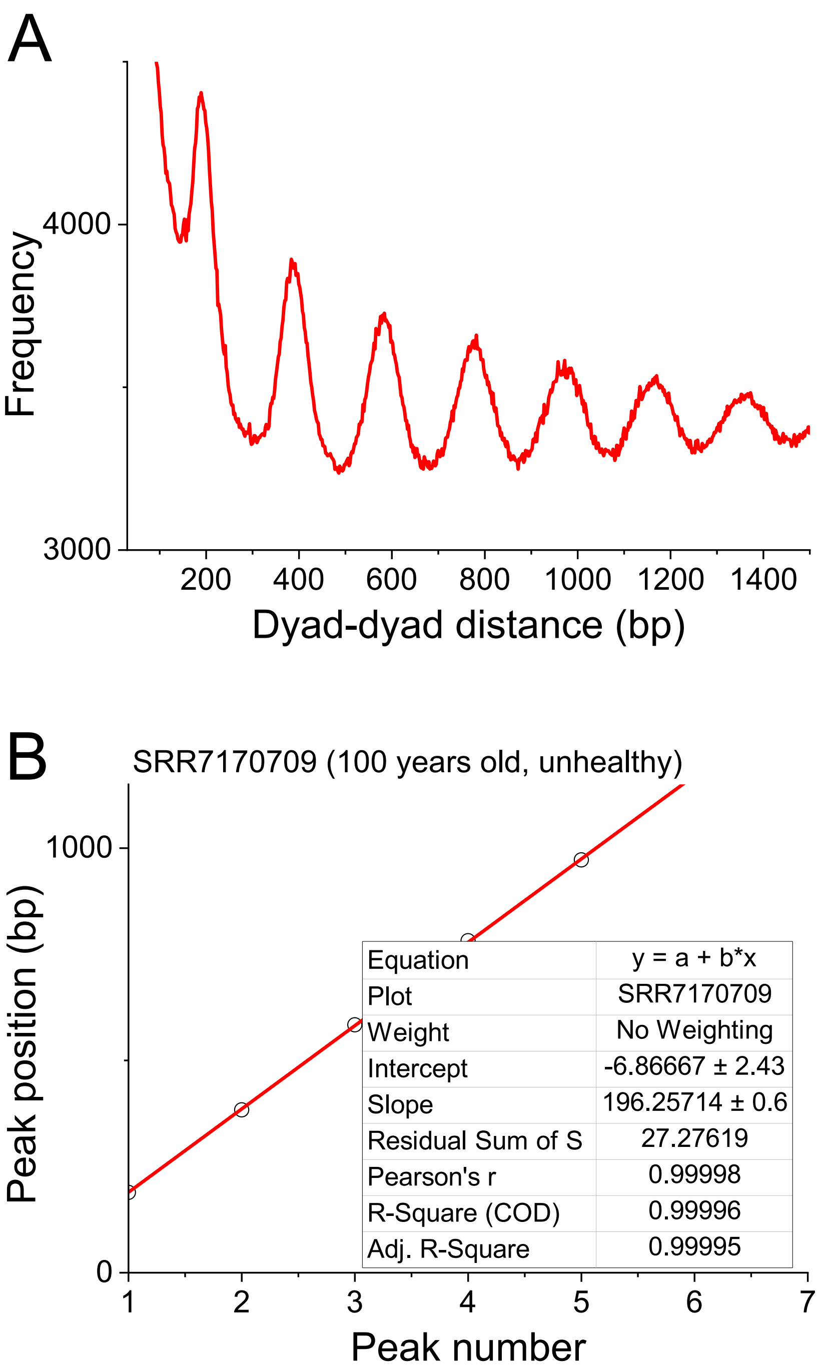

Supplementary Figure S12. A) The distribution of distances between centers of cfDNA fragments calculated with NucTools for cfDNA sample from a 100 years old person (SRA accession number SRR7170709), averaged across all chromosomes. B) Linear regression of the locations of the peak summits of the average genome-wide profile of the distribution of distances between centers of cfDNA fragments from (A). The slope of the linear fit line is equal to the nucleosome repeat length value.

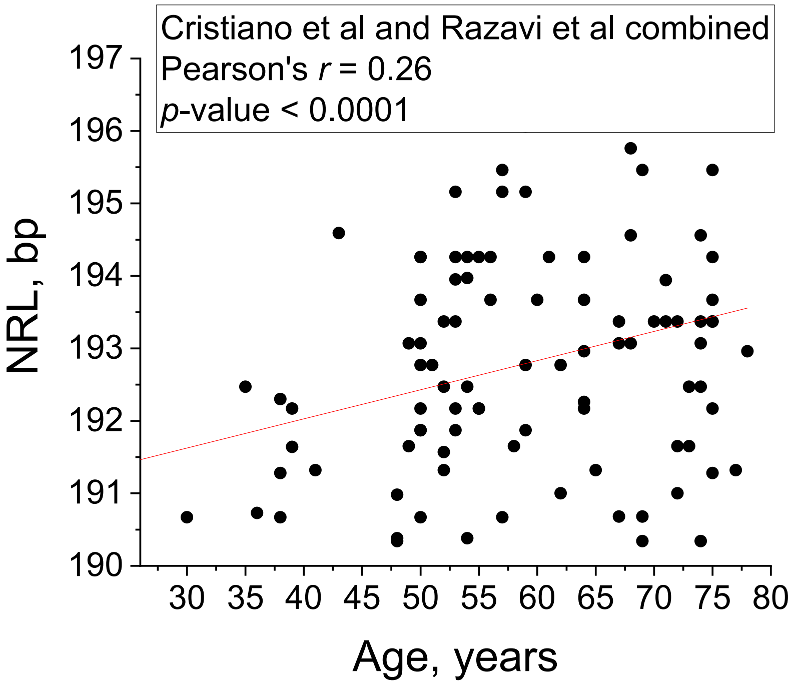

Supplementary Figure S13. Correlation between NRL and age for a combined dataset composed of deep-sequenced cfDNA samples from Cristiano et al (N=79) and Razavi et al (N=24).

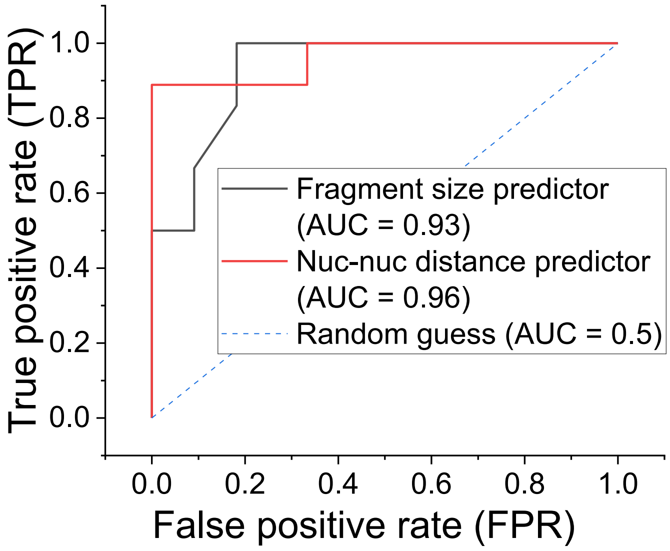

Supplementary Figure S14. Receiver-Operator Curves for the classification of cfDNA samples into those belonging to people ≤ 55 y.o. and >55 y.o. Black – predictor based on cfDNA fragment sizes (AUC = 0.93). Red – predictor based on nucleosome-nucleosome distances (AUC = 0.96). Blue dashed line – random guess (AUC = 0.5). The analysis is performed using the Cristiano et al dataset, as in Figure 2.

Table S1. NRL values calculated for the dataset of Cristiano et al.

Sample ID	NRL, bp	Error, bp	Age, years
EGAR00002028008_PGDX18436P	193.95	1.2	53
EGAR00002027976_PGDX18344P	194.26	0.2	56
EGAR00002027973_PGDX18341P	195.16	1.2	59
EGAR00002027939_PGDX17947P	191.87	1.2	50
EGAR00002027935_PGDX17940P	193.07	0.2	68
EGAR00002027920_PGDX17953P	195.46	0.2	69
EGAR00002028067_PGDX18712P	192.47	0.2	54
EGAR00002028060_PGDX18706P	195.16	1.2	57
EGAR00002028059_PGDX18705P	191.57	1.2	52
EGAR00002028058_PGDX18704P	193.37	1.2	75
EGAR00002028057_PGDX18703P	190.68	1.2	67
EGAR00002028055_PGDX18702P	193.67	1.2	60
EGAR00002028038_PGDX18689P	193.37	1.2	74
EGAR00002028035_PGDX18460P	193.67	1.2	64
EGAR00002027899_PGDX18285P	192.17	1.2	55
EGAR00002027892_PGDX18276P	193.37	1.2	71
EGAR00002027881_PGDX18265P	192.17	1.2	75
EGAR00002027874_PGDX17942P	194.26	0.2	75
EGAR00002027862_PGDX17972P	192.26	1.2	64
EGAR00002027859_PGDX17970P	191.87	1.2	59
EGAR00002027858_PGDX17990P	193.07	1.2	50
EGAR00002027851_PGDX16593P	195.46	1.2	75
EGAR00002027850_PGDX16592P	194.56	1.2	74
EGAR00002027846_PGDX16586P	192.47	1.2	73
EGAR00002027841_PGDX16581P	196.36	1.2	74
EGAR00002027840_PGDX16580P	192.17	1.2	64
EGAR00002027837_PGDX16570P	193.07	1.2	74
EGAR00002027836_PGDX16569P	193.37	1.2	67
EGAR00002027835_PGDX16568P	192.47	1.2	74
EGAR00002027839_PGDX16579P	191.28	1.2	75
EGAR00002027848_PGDX16588P	197.25	0.2	68
EGAR00002027849_PGDX16591P	193.67	1.2	75
EGAR00002027852_PGDX16594P	193.37	1.2	75
EGAR00002027856_PGDX17986P	194.26	1.2	61
EGAR00002027857_PGDX17963P	194.26	1.2	55
EGAR00002027861_PGDX18288P	192.77	1.2	59
EGAR00002027866_PGDX17968P	194.26	0.2	53
EGAR00002027869_PGDX17958P	193.07	1.2	67
EGAR00002027890_PGDX4549P	190.38	1.2	48
EGAR00002027898_PGDX18284P	192.47	1.2	52
EGAR00002027900_PGDX4550P	190.73	1.2	36
EGAR00002027903_PGDX17944P	193.37	1.2	72
EGAR00002027905_PGDX17941P	196.66	1.2	50
EGAR00002027910_PGDX17951P	192.17	1.2	55
EGAR00002028021_PGDX4562P	191.28	0.2	38
EGAR00002028031_PGDX4563P	191.64	1.2	39
EGAR00002028036_PGDX18461P	192.17	1.2	53
EGAR00002028041_PGDX18691P	194.26	0.2	54
EGAR00002028042_PGDX18692P	193.37	1.2	52
EGAR00002028043_PGDX4564P	188.59	1.2	55

EGAR00002028051_PGDX18699P	192.77	1.2	51
EGAR00002028053_PGDX18701	189.18	1.2	54
EGAR00002028056_PGDX4565P	190.98	1.2	48
EGAR00002028064_PGDX18710P	193.97	1.2	54
EGAR00002028066_PGDX18711P	194.26	0.2	50
EGAR00002027912_PGDX4551P	192.17	1.2	39
EGAR00002027913_PGDX17946P	192.17	1.2	50
EGAR00002027915_PGDX18326P	195.16	1.2	53
EGAR00002027917_PGDX18328P	193.37	1.2	53
EGAR00002027918_PGDX18329P	192.77	1.2	62
EGAR00002027959_PGDX4556P	190.38	1.2	54
EGAR00002027969_PGDX4557P	193.07	0.2	49
EGAR00002027970_PGDX18338P	190.68	1.2	69
EGAR00002027980_PGDX18348P	193.67	1.2	50
EGAR00002027981_PGDX18349P	196.06	1.2	59
EGAR00002027989_PGDX4559P	189.18	1.2	58
EGAR00002027993_PGDX17981P	191.87	1.2	53
EGAR00002028000_PGDX4560P	192.47	0.2	35
EGAR00002028010_PGDX4561P	191.87	1.2	50

Table S2. NRL values calculated for the dataset of Peneder et al

Sample ID	Age, years	NRL, bp	Error, bp
1	37	194.05	0.4
2	44	195.1	1.1
3	48	194.1	0.7
4	40	195.1	0.8
5	50	194.8	1.1
6	25	194	1
7	24	192.1	0.2
8	32	193	1
9	37	192.5	0.4
10	29	194.4	0.8
11	31	195.1	1
12	27	191	0.4
13	30	193.2	0.3
14	27	195.3	0.7
15	36	194.1	0.8
16	25	192.1	0.7
17	39	192.9	0.6
19	40	190.6	0.6
20	30	192.1	0.4
21	32	192	0.6
22	31	192	0.8
18	34	192.9	0.5

Table S3. NRL values calculated for the dataset of Razavi et al.

Sample ID	Age, years	NRL, bp	Error, bp
EGAZ00001444737_SDBBW044216563294CH1	30	190.67	0.2
EGAZ00001444738_SDBBW044216563299CH	71	193.94	0.2
EGAZ00001444739_SDBBW044216563300CH	72	191.65	1.2
EGAZ00001444740_SDBBW044216563303CH	48	190.34	1.2
EGAZ00001444741_SDBBW044216563304CH	58	191.65	1.2
EGAZ00001444742_SDBBW044216563305CH	38	192.3	1.2
EGAZ00001444743_SDBBW044216563328CH	77	191.32	1.2
EGAZ00001444744_SDBBW044216563329CH	72	191	1.2
EGAZ00001444746_SDBBW044216563335CH	74	190.34	1.2
EGAZ00001444747_SDBBW044216563342CH	20	189.04	1.2
EGAZ00001444748_SDBBW044216563343CH	50	190.67	0.2
EGAZ00001444749_SDBBW044216563344CH	78	192.96	1.2
EGAZ00001444750_SDBBW044216563354CH	49	191.65	1.2
EGAZ00001444751_SDBBW044216563359CH	62	191	1.2
EGAZ00001444752_SDBBW044216563361CH	73	191.65	1.2
EGAZ00001444753_SDBBW044216563364CH	41	191.32	0.2
EGAZ00001444754_SDBBW044216563365CH	57	190.67	0.2
EGAZ00001444755_SDBBW044216563366CH	43	194.59	1.2
EGAZ00001444756_SDBBW044216563367CH	69	190.34	1.2
EGAZ00001444757_SDBBW044216563368CH	64	192.96	1.2
EGAZ00001444758_SDBBW044216563370CH	63	189.69	1.2
EGAZ00001444759_SDBBW044216563371CH	38	190.67	0.2
EGAZ00001444760_SDBBW044216563375CH	65	191.32	0.2

Supplementary references

Piroeva, K. V., McDonald, C., Xanthopoulos, C., Fox, C., Clarkson, C. T., Mallm, J.-P., . . . Teif, V. B. (2023). Nucleosome repositioning in chronic lymphocytic leukaemia. *Genome Res, 33*, 1649-1661. doi:10.1101/gr.277298.122

Quinlan, A. R., & Hall, I. M. (2010). BEDTools: a flexible suite of utilities for comparing genomic features. *Bioinformatics, 26*(6), 841-842. doi:10.1093/bioinformatics/btq033
